# Supplementary material for: Differentially Expressed Candidate miRNAs of Day 16 Bovine Embryos on the Regulation of Pregnancy Establishment in Dairy Cows
Source: Animals (Basel). 2023 Sep 28;13(19):3052. doi: 10.3390/ani13193052 (PMC10571895; doi:10.3390/ani13193052)
Supplement: Supplementary file 1 [file animals-13-03052-s001.zip › animals-2614912-supplementary/FigureS1.docx]

**Figure S1**. The ethidium bromide-stained [electrophoresis gel](https://www.sciencedirect.com/topics/agricultural-and-biological-sciences/gel-electrophoresis), with [amplicons](https://www.sciencedirect.com/topics/agricultural-and-biological-sciences/amplicon) of expected sizes


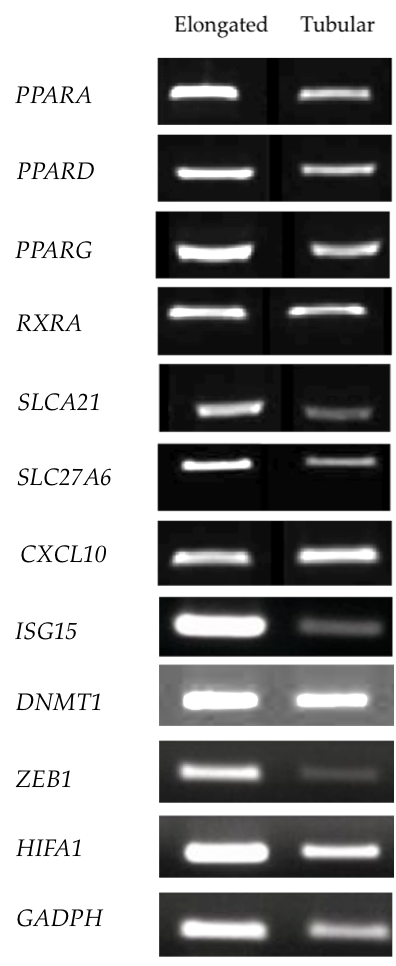


Elongated conceptus - ≥25 mm long; Tubular conceptus - 10 to 20 mm long.

*PPAR* - peroxisome proliferator-activated receptor; *RXR* - retinoid X receptor; *SLC2A1* - Solute Carrier Family 2 Member 1; *SLC27A6* - Solute Carrier Family 27 Member 6; *CXCL10* - C-X-C Motif Chemokine Ligand 10; *ISG15* - interferon-stimulated gene-15; *DNMT*1 - DNA methyltransferase 1; *ZEB1* - Zinc Finger E-Box Binding Homeobox 1; *HIF1A* - Hypoxia inducing factor 1A; *GAPDH* - glyceraldehyde 3-phosphate dehydrogenase.
