# Supplementary material for: Differentially Expressed Candidate miRNAs of Day 16 Bovine Embryos on the Regulation of Pregnancy Establishment in Dairy Cows
Source: Animals (Basel). 2023 Sep 28;13(19):3052. doi: 10.3390/ani13193052 (PMC10571895; doi:10.3390/ani13193052)
Supplement: Supplementary file 1 [file animals-13-03052-s001.zip › animals-2614912-supplementary/FigureS2.docx]

**Figure S2.** Immunoblot lanes of protein of candidate genes in day 16 embryos.


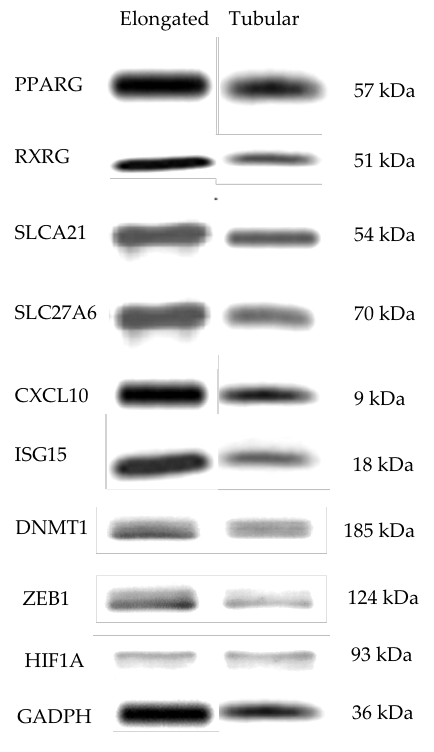


Elongated conceptus - ≥25 mm long; Tubular conceptus - 10 to 20 mm long.

PPAR - peroxisome proliferator-activated receptor; RXRG - retinoid X receptor gamma; SLC2A1 - Solute Carrier Family 2 Member 1; SLC27A6 - Solute Carrier Family 27 Member 6; CXCL1- C-X-C Motif Chemokine Ligand 10; ISG15 - interferon-stimulated gene-15; DNA methyltransferase 1; ZEB1, Zinc Finger E-Box Binding Homeobox 1; Hypoxia inducing factor 1A (HIF1A); GAPDH - glyceraldehyde 3-phosphate dehydrogenase.
