# Supplementary material for: Differentially Expressed Candidate miRNAs of Day 16 Bovine Embryos on the Regulation of Pregnancy Establishment in Dairy Cows
Source: Animals (Basel). 2023 Sep 28;13(19):3052. doi: 10.3390/ani13193052 (PMC10571895; doi:10.3390/ani13193052)
Supplement: Supplementary file 1 [file animals-13-03052-s001.zip › animals-2614912-supplementary/TableS1.docx]

Table S1. Bovine miRBase profiler plate, consisting of primers for 84 target miRNAs and control genes.

| Layout | 1 | 2 | 3 | 4 | 5 | 6 | 7 | 8 | 9 | 10 | 11 | 12 |
| --- | --- | --- | --- | --- | --- | --- | --- | --- | --- | --- | --- | --- |
| A | bta-let-7f | bta-miR-101 | bta-miR-103 | bta-miR-125a | bta-miR-125b | bta-miR-126-3p | bta-miR-128 | bta-miR-145 | bta-miR-148a | bta-miR-151-3p | bta-miR-151-5p | bta-miR-16b |
| B | bta-miR-181a | bta-miR-18a | bta-miR-18b | bta-miR-199a-5p | bta-miR-205 | bta-miR-20a | bta-miR-21-5p | bta-miR-221 | bta-miR-222 | bta-miR-26a | bta-miR-26b | bta-miR-27a-3p |
| C | bta-miR-27b | bta-miR-29a | bta-miR-300-5p | bta-miR-30d | bta-miR-31 | bta-miR-320a | bta-miR-34b | bta-miR-484 | bta-miR-499 | bta-miR-99a-5p | bta-miR-7a-5p | bta-let-7d |
| D | bta- let-7g | bta- let-7i | bta-miR-17-5p | bta-miR-107 | bta-miR-10a | bta-miR-10b | bta-miR-122 | bta-miR-124b | bta-miR-127 | bta-miR-132 | bta-miR-138 | bta-miR-139 |
| E | bta-miR-140 | bta-miR-142-3p | bta-miR-142-5p | bta-miR-148b | bta-miR-150 | bta-miR-15b | bta-miR-17-3p | bta-miR-17-5p | bta-miR-181b | bta-miR-181c | bta-miR-186 | bta-miR-191 |
| F | bta-miR-192 | bta-miR-193a-3p | bta-miR-193a-5p | bta-miR-199a-3p | bta-miR-199b | bta-miR-200a | bta-miR-200b | bta-miR-200c | bta-miR-20b | bta-miR-210 | bta-miR-21-3p | bta-miR-214 |
| G | bta-miR-215 | bta-miR-218 | bta-miR-22-5p | bta-miR-23a | bta-miR-23b-3p | bta-miR-24-3p | bta-miR-25 | bta-miR-29b | bta-miR-29c | bta-miR-30a-5p | bta-miR-30c | bta-miR-30e-5p |
| H | cel-miR39-3p | cel-miR39-3p | SNORD42B | SNORD69 | SNORD61 | SNORD68 | SNORD96A | RNU6-6P | miRTC | miRTC | PPC | PPC |

Characterized 84 target miRNAs (plate well positions A1 - G12) and controls (plate well positions H1 - H12).
