# Supplementary material for: Differentially Expressed Candidate miRNAs of Day 16 Bovine Embryos on the Regulation of Pregnancy Establishment in Dairy Cows
Source: Animals (Basel). 2023 Sep 28;13(19):3052. doi: 10.3390/ani13193052 (PMC10571895; doi:10.3390/ani13193052)
Supplement: Supplementary file 1 [file animals-13-03052-s001.zip › animals-2614912-supplementary/TableS2.docx]

**Table S2.** Forward and reverse primers sequence for quantitative real-time polymerase chain reaction amplification of mRNA for day 16 bovine conceptus samples

| Gene | Primer sequence (5’–3’) | Product length (bp) | Accession number |
| --- | --- | --- | --- |
| *PPARA* | F: GCCCCAGGTGGTGGA  R: CCGGCCACAGACTGTTACTT | 122 | NM_001034036.1 |
| *PPARD* | F: AGTGGCTTCTGTTCACCGAC  R: GCTGGAAGGAAGTGAGTGCT | 266 | NM_001083636.1 |
| *PPARG* | F: CACAGAGATGCCGTTTTGGC  R: CAACCATCGGGTCAGCTCTT | 173 | NM_181024.2 |
| *RXRA* | F: CCTTGACTGCCAGGACTTCTCC  R: GGGGGAACTGATGACCGAGA | 214 | XM_024998424.1 |
| *RXRB* | F: GGAGCCATCTTCGATAGGGT  R: GCCTATGGACCTGAGAGCAG | 261 | NM_001083640.1 |
| *RSAD2* | F: GCTGGTACCCATTGCGTTTG  R: CTGGCGGGTGAAGTGGTAAT | 244 | NM_001045941.1 |
| *SLC2A1* | F: CTCATAGCCTGCATCTCGCA  R: CCTGTTCCGGAGAGCATTGT | 237 | NM_174829.3 |
| *SLC27A6* | F: TGGAGCACGCAGTGATGTAT  R: AAGTCCGGGTTCCCCTTTTT | 256 | NM_001101169.1 |
| *CXCL10* | F: CTGCCCACGTGTCGAGATTA  R: AAACCGAAGTCCACGGACAA | 251 | NM_001046551.2 |
| *ISG15* | F: TGTCTTTTGAAGGGAGGCCC  R: TTATTCACTGCGCTGCATGG | 156 | NM_174366.1 |
| *DNMT1* | F: TATCGGCTGTTCGGCAACAT  R: GGCAGCCTCCTCCTTGATTT | 153 | NM_182651.2 |
| *ZEB1* | F: AAAGCAGCAGGGCGAGTTAT  R: TATGGGGTTGGCACTTGGTG | 181 | NM_001206590.1 |
| *HIF1A* | F: GCATCGCGGGCACCGATTCACCAT | 165 | NM_174339.3 |
|  | R: TGGGGGAGTGGCAACTGATGAGCA |  |  |
| *GADPH* | F: GTGAAGGTCGGAGTGAACGG | 93 | NM_001034034.2 |
|  | R: ATTGATGGCGACGATGTCCA |  |  |

*PPAR* - Peroxisome proliferator-activated receptor; *RXR* - Retinoid X receptor; *SLC2A1* - Solute Carrier Family 2 Member 1; *SLC27A6* - Solute Carrier Family 27 Member 6; *CXCL10* - C-X-C motif chemokine ligand 10; *ISG15* - Interferon interferon-stimulated gene-15; *DNMT1* - DNA methyltransferase 1; *ZEB1* - Zinc Finger E-Box Binding Homeobox 1; *HIF1A* - Hypoxia inducing factor 1A; *GAPDH* - glyceraldehyde 3-phosphate dehydrogenase.
