# Supplementary material for: Differentially Expressed Candidate miRNAs of Day 16 Bovine Embryos on the Regulation of Pregnancy Establishment in Dairy Cows
Source: Animals (Basel). 2023 Sep 28;13(19):3052. doi: 10.3390/ani13193052 (PMC10571895; doi:10.3390/ani13193052)
Supplement: Supplementary file 1 [file animals-13-03052-s001.zip › animals-2614912-supplementary/TableS3.docx]

**Table S3.** Primary, peptide or proteins and secondary antibodies used for determination of proteins

| **Targets** | **Primary Antibody** | **Secondary Antibody** |
| --- | --- | --- |
| PPARG | Rabbit anti-PPARG polyclonal (AV32880)§ | Mouse anti-rabbit IgG-FITC (sc-2359) |
| RXRG | Rabbit anti-RXRG polyclonal (AV 45631)§ | Mouse anti-rabbit IgG-FITC (sc-2359) |
| SLC2A1 | Rabbit anti-SLC2A1(GLUT1) polyclonal (PA1-1063)Ø | Mouse anti-rabbit IgG-FITC (sc-2359) |
| SLC27A6 | Rabbit anti-SLC27A6 polyclonal (SAB2102195)§ | Mouse anti-rabbit IgG-FITC (sc-2359) |
| CXCL10 | Rabbit anti-CXCL10 polyclonal (PB0385B-100)‡ | Mouse anti-rabbit IgG-FITC (sc-2359) |
| ISG15 | Rabbit anti-ISG15 monoclonal (7H29L24)† | Mouse anti-rabbit IgG-FITC (sc-2359) |
| DNMT1 | Mouse monoclonal antibody (60B1220.1)† | Goat anti-Mouse IgG H&L-FITC (ab6785) |
| ZEB1 | Mouse monoclonal antibody (14-9741-82)Ø | Goat anti-Mouse IgG H&L-FITC (ab6785) |
| HIF1A | Mouse monoclonal antibody (sc-53546)† | Goat anti-Mouse IgG H&L-FITC (ab6785) |
| GAPDH | Mouse anti-GAPDH monoclonal (sc-166545) | Goat anti-Mouse IgG H&L-FITC (ab6785) |

§Sigma-Aldrich; ØThermo Fisher Scientific; ‡Kingfisher Biotech Inc.; †Invitrogen; sc – SantaCruz Inc.; Ab – Abcam;

PPAR - peroxisome proliferator-activated receptor; RXRG - retinoid X receptor gamma; SLC2A1 - Solute Carrier Family 2 Member 1; SLC27A6 - Solute Carrier Family 27 Member 6; CXCL1- C-X-C Motif Chemokine Ligand 10; ISG15 - interferon-stimulated gene-15; DNMT1, DNA methyltransferase 1; ZEB1, Zinc Finger E-Box Binding Homeobox 1; Hypoxia inducing factor 1A (HIF1A); GAPDH - glyceraldehyde 3-phosphate dehydrogenase.
