# Supplementary material for: Differentially Expressed Candidate miRNAs of Day 16 Bovine Embryos on the Regulation of Pregnancy Establishment in Dairy Cows
Source: Animals (Basel). 2023 Sep 28;13(19):3052. doi: 10.3390/ani13193052 (PMC10571895; doi:10.3390/ani13193052)
Supplement: Supplementary file 1 [file animals-13-03052-s001.zip › animals-2614912-supplementary/TableS4.docx]

**Table S4.** Nucleotide sequences of cattle and human miRNAs (www.mirbase.org)

| Bovine miRNA ID | | Human miRNA | |
| --- | --- | --- | --- |
| ID | Sequence | Sequence | ID |
| bta-mir-107 | AGCAGCAUUGUACAGGGCUAUC | AGCAGCAUUGUACAGGGCUAUCA | hsa-mir-107 |
| bta-mir-124-3p | UCACAGUGAACCGGUCUCUUU | UCACAGUGAACCGGUCUCUUU | hsa-mir-128-3p |
| bta-mir-128-3p | AGCUGGUGUUGUGAAUCAGGCCG | AGCUGGUGUUGUGAAUCAGGCCG | hsa-mir-138-5p |
| bta-mir-138-5p | ACUGCAGUGAAGGCACUUGU | ACUGCAGUGAAGGCACUUGUAG | hsa-mir-17-3p |
| bta-mir-17-3p | CAAAGUGCUUACAGUGCAGGUAGU | CAAAGUGCUUACAGUGCAGGUAG | hsa-mir-17-5p |
| bta-mir-17-5p | AACAUUCAUUGCUGUCGGUGGGUU | AACAUUCAUUGCUGUCGGUGGGU | hsa-mir-181b-5p |
| bta-miR-181b-1 | CAAAGAAUUCUCCUUUUGGGCU | CAAAGAAUUCUCCUUUUGGGCU | hsa-mir-186-5p |
| bta-mir-191-5p | CAACGGAAUCCCAAAAGCAGCUG | CAACGGAAUCCCAAAAGCAGCUG | hsa-mir-191-5p |
| bta-miR-199a-5p | CCCAGUGUUCAGACUACCUGUU | CCCAGUGUUCAGACUACCUGUUC | hsa-mir-199a-5p |
| bta-miR-200-3p | UAAUACUGCCUGGUAAUGAUG | UAAUACUGCCUGGUAAUGAUGA | hsa-mir-200b-3p |
| bta-miR-210-3p | ACUGUGCGUGUGACAGCGGCUGA | CUGUGCGUGUGACAGCGGCUGA | hsa-mir-210-3p |
| bta-mir-218-5p | UUGUGCUUGAUCUAACCAUGUG | UUGUGCUUGAUCUAACCAUGU | hsa-mir-218-5p |
| bta-miR-25-3p | CAUUGCACUUGUCUCGGUCUGA | CAUUGCACUUGUCUCGGUCUGA | hsa-mir-25-3p |
| bta-miR-26a-5p | UUCAAGUAAUCCAGGAUAGGCU | UUCAAGUAAUCCAGGAUAGGCU | hsa-mir-26a-5p |
| bta-miR-26b-3p | UUCAAGUAAUUCAGGAUAGGUU | UUCAAGUAAUUCAGGAUAGGU | hsa-mir-26b-5p |
| bta-mir-30d | UGUAAACAUCCCCGACUGGAAGCU | UGUAAACAUCCCCGACUGGAAG | hsa-mir-30d-5p |
| bta-mir-30e | CUUUCAGUCGGAUGUUUACAGC | CUUUCAGUCGGAUGUUUACAGC | hsa-mir-30e-3p |
| bta-mir-320a-1 | AAAAGCUGGGUUGAGAGGGCGA | AAAAGCUGGGUUGAGAGGGCGA | hsa-mir-320a |
| bta-mir-484 | UCAGGCUCAGUCCCCUCCCGAU | UCAGGCUCAGUCCCCUCCCGAU | hsa-mir-484 |

Note: Nucleotide sequences are conserved among human, mouse and cattle.

Pink color denote difference in sequences.
