# Supplementary material for: Differentially Expressed Candidate miRNAs of Day 16 Bovine Embryos on the Regulation of Pregnancy Establishment in Dairy Cows
Source: Animals (Basel). 2023 Sep 28;13(19):3052. doi: 10.3390/ani13193052 (PMC10571895; doi:10.3390/ani13193052)
Supplement: Supplementary file 1 [file animals-13-03052-s001.zip › animals-2614912-supplementary/TableS5.docx]

**Table S5.** Hub genes, their associated conserved miRNAs and annotation terms

| **Hub genes** | **Associated miRNAs** | **GO annotation terms** |
| --- | --- | --- |
| BMI1 | hsa-mir-218-5p, hsa-mir-200b-3p, hsa-mir-128-3p, hsa-mir-320a, hsa-mir-30e-5p, hsa-mir-186-5p, hsa-mir-30d-5p, hsa-mir-30e-5p | in utero embryonic development; embryonic skeletal system morphogenesis; zinc binding; chromatin remodeling; positive regulation of immature T cell proliferation; positive regulation of ubiquitin-protein transferase activity; apoptotic signaling pathway; negative regulation of transcription by RNA polymerase II |
| CCND1 | hsa-mir-17-5p, hsa-mir-26b-5p, hsa-mir-138-5p, hsa-mir-107, hsa-mir-128-3p, hsa-mir-186-5p, hsa-mir-200b-3p, hsa-mir-218-5p, hsa-mir-25-3p, hsa-mir-30d-5p, hsa-mir-191-5p, hsa-mir-30e-5p | G1/S transition of mitotic cell cycle; cell division; response to Leptin; negative regulation of transcription by RNA polymerase II; positive regulation of G2/M transition of mitotic cell cycle; DNA damage response |
| CCND2 | hsa-mir-17-5p, hsa-mir-26a-5p, hsa-mir-26b-5p  hsa-mir-191-5p, hsa-mir-320a, hsa-mir-17-3p, hsa-mir-186-5p, hsa-mir-210-3p, hsa-mir-218-5p, hsa-mir-25-3p, hsa-mir-107, hsa-mir-128-3p, hsa-mir-30e-3p | positive regulation of cell population proliferation; negative regulation of apoptotic process; G1/S transition of mitotic cell cycle; regulation of cyclin-dependent protein serine/threonine kinase activity; mitotic cell cycle phase transition |
| CDKN1A | hsa-mir-17-5p, hsa-mir-107, hsa-mir-128-3p, hsa-mir-17-3p, hsa-mir-186-5p, hsa-mir-218-5p, hsa-mir-25-3p, hsa-mir-26a-5p, hsa-mir-26b-5p, hsa-mir-191-5p, hsa-mir-200b-3p, hsa-mir-210-3p, hsa-mir-181b-5p | cyclin-dependent protein serine/threonine kinase inhibitor activity; ubiquitin protein ligase binding; protein transport; DNA damage response; cellular response to amino acid starvation; regulation of cell growth; regulation of programmed cell death; regulation of reactive oxygen species metabolic process; regulation of vascular associated smooth muscle cell proliferation; regulation of G1/S transition of mitotic cell cycle |
| CDKN1B | hsa-mir-218-5p, hsa-mir-200b-3p, hsa-mir-30e-3p, hsa-mir-17-5p, hsa-mir-186-5p, hsa-mir-25-3p, hsa-mir-191-5p | protein phosphatase binding; placenta development; potassium ion transport; NITCH signaling pathway; protein kinase bunding; autophagy; regulation cell proliferation and migration; regulation of G1/S transition of mitotic cell cycle |
| CDK6 | hsa-mir-26a-5p, hsa-mir-26b-5p, hsa-mir-107, hsa-mir-218-5p, hsa-mir-191-5p, hsa-mir-320a, hsa-mir-128-3p, hsa-mir-186-5p,hsa-mir-25-3p, hsa-mir-30d-5p, hsa-mir-138-5p, hsa-mir-30e-3p, hsa-mir-30e-5p | negative regulation of transcription by RNA polymerase II; T cell differentiation; positive regulation of gene expression; cell dedifferentiation; regulation of hematopoietic stem cell differentiation; cyclin-dependent protein serine/threonine kinase activity; protein phosphorylation; |
| DICER1 | hsa-mir-107, hsa-mir-218-5p, hsa-mir-128-3p, hsa-mir-17-3p, hsa-mir-17-5p, hsa-mir-186-5p, hsa-mir-199a-5p, hsa-mir-25-3p, hsa-mir-26a-5p, hsa-mir-26b-5p, hsa-mir-30d-5p, hsa-mir-30e-3p | pre-miRNA binding; global gene silencing by mRNA cleavage; NIK/NF-kappaB signaling; negative regulation of TNF production; RISC complex; apoptotic DNA fragmentation |
| DNMT1 | hsa-mir-17-5p, hsa-mir-26a-5p, hsa-mir-218-5p, hsa-mir-200b-3p, hsa-mir-17-3p, hsa-mir-107 | post-fertilization epigenetic regulation of gene expression; zinc ion binding; DNA binding; promoter-specific chromatin binding; cellular response to amino acid stimulus; regulation of vascular associated smooth muscle cell proliferation |
| E2F1 | hsa-mir-17-5p, hsa-mir-181b-5p, hsa-mir-107, hsa-mir-17-3p, hsa-mir-320a | DNA-binding transcription factor binding; DNA damage checkpoint signaling; regulation of apoptotic process; fibroblast proliferation; brain development; G1/S transition of mitotic cell cycle |
| E2F3 | hsa-mir-17-5p, hsa-mir-199a-5p, hsa-mir-210-3p, hsa-mir-200b-3p, hsa-mir-128-3p, hsa-mir-107, hsa-mir-186-5p, hsa-mir-25-3p, hsa-mir-26a-5p, hsa-mir-26b-5p, hsa-mir-30d-5p, hsa-mir-320a, hsa-mir-191-5p, hsa-mir-30e-3p, hsa-mir-30e-5p | Protein transport; positive regulation of transcription by RNA polymerase II; G1/S transition of mitotic cell cycle; positive regulation of cell population proliferation; negative regulation of fat cell proliferation; positive regulation of vascular associated smooth muscle cell apoptotic process; DNA-binding transcription factor activity, RNA polymerase II-specific |
| ESR1 | hsa-mir-26a-5p, hsa-mir-17-5p, hsa-mir-191-5p, hsa-mir-320a | TFIIB-class transcription factor binding; chromatin, zinc, β-catenin, steroid and protein binding; follicle growth, ESR binding/activity; regulation of cytosolic calcium ion concentration; gonad, reproductive tract and mammary gland development; cellular response; regulation of nitric oxide biosynthetic process; stem cell differentiation; |
| EZH2 | hsa-mir-25-3p, hsa-mir-26a-5p, hsa-mir-26b-5p, hsa-mir-199a-5p, hsa-mir-30d-5p, hsa-mir-200b-3p, hsa-mir-320a, hsa-mir-138-5p, hsa-mir-128-3p, hsa-mir-107 | chromatin binding; heterochromatin formation; histone H3K27 methyltransferase activity; |
| FOXO1 | hsa-mir-107, hsa-mir-186-5p, hsa-mir-17-3p, hsa-mir-17-5p, hsa-mir-200b-3p, hsa-mir-210-3p | positive regulation of gluconeogenesis; insulin receptor signaling pathway; cellular response to hyperoxia; autophagy; apoptotic process; regulation of transcription by RNA polymerase II |
| HIF1A | hsa-mir-17-5p, hsa-mir-107, hsa-mir-199a-5p, hsa-mir-210-3p, hsa-mir-138-5p, hsa-mir-186-5p, hsa-mir-218-5p, hsa-mir-128-3p | response to reactive oxygen species; response to hypoxia; intracellular glucose homeostasis; regulation of glycolytic process; positive regulation of VEGF production; regulation of transcription by RNA polymerase II |
| MCL1 | hsa-mir-17-5p, hsa-mir-26a-5p, hsa-mir-26b-5p, hsa-mir-30d-5p, hsa-mir-181b-5p, hsa-mir-186-5p, hsa-mir-320a, hsa-mir-128-3p, hsa-mir-200b-3p, hsa-mir-25-3p, hsa-mir-191-5p, hsa-mir-210-3p, hsa-mir-30e-3p, hsa-mir-30e-5p | cell differentiation; transmembrane transport; regulation of mitochondrial function; extrinsic apoptotic signaling pathway |
| MDM2 | hsa-mir-17-5p, hsa-mir-25-3p, hsa-mir-26a-5p, hsa-mir-26b-5p, hsa-mir-218-5p, hsa-mir-107, hsa-mir-17-3p, hsa-mir-186-5p, hsa-mir-210-3p, hsa-mir-30d-5p, hsa-mir-30e-3p, hsa-mir-30e-5p | zinc binding; ubiquitin binding; apoptotic process; protein ubiquitination; cellular response to hypoxia; negative regulation of transcription by RNA polymerase II; blood vessel development; |
| MYC | hsa-mir-17-5p, hsa-mir-25-3p, hsa-mir-26a-5p, hsa-mir-30d-5p, hsa-mir-186-5p, hsa-mir-320a, hsa-mir-30e-3p, hsa-mir-107, hsa-mir-138-5p, hsa-mir-17-3p, hsa-mir-199a-5p, hsa-mir-26b-5p, hsa-mir-191-5p, hsa-mir-200b-3p, hsa-mir-30e-3p, hsa-mir-30e-5p | DNA-binding transcription factor activity, RNA polymerase II-specific; transcription coregulator binding; DNA binding; protein dimerization; G1/S transition of mitotic cell cycle; chromatin remodeling; DNA damage response; regulations of cell cycle, cellular response to hypoxia, DNA binding, telomere maintenance, apoptotic process, and epithelial cell, somatic stem cell and fibroblast proliferation |
| NOTCH1 | hsa-mir-30d-5p, hsa-mir-200b-3p, hsa-mir-128-3p, hsa-mir-17-3p, hsa-mir-30e-5p | *in utero* embryonic development; sprouting angiogenesis; organ development; transmembrane signaling receptor, transcription coactivator, and enzyme inhibitor activity; Ca ion, enzyme, and chromatin DNA binding; humoral immune response; |
| PTEN | hsa-mir-17-5p, hsa-mir-17-3p, hsa-mir-25-3p, hsa-mir-26a-5p, hsa-mir-26b-5p, hsa-mir-107, hsa-mir-181b-5p, hsa-mir-128-3p, hsa-mir-320a, hsa-mir-210-3p, hsa-mir-30e-3p | phosphatidylinositol 3-kinase signaling; cell motility; plasma membrane; regulation of cell population proliferation |
| ZEB1 | hsa-mir-200b-3p, hsa-mir-26a-5p, hsa-mir-128-3p, hsa-mir-210-3p | DNA-binding transcription factor activity, RNA polymerase II-specific; chromatin binding; regulation of TGFRB pathway; regulation of T cell differentiation; nervous system development; anatomical structure development; embryo organ morphogenesis |
